# Role of Acetaldehyde in Mediating the Pharmacological and Behavioral Effects of Alcohol

**Published:** 2006

**Authors:** Etienne Quertemont, Vincent Didone

**Affiliations:** Etienne Quertemont, Ph.D., is an associate professor and Vincent Didone is a research assistant in the Centre de Neurosciences Cognitives et Comportementales, Université de Liège, Liège, Belgium

**Keywords:** Ethanol metabolism, ethanol-to-acetaldehyde metabolism, acetaldehyde, aldehyde dehydrogenases (ALDHs), alcohol dehydrogenase (ADH), alcohol metabolite, catalase, brain, central nervous system, protective factors, alcohol flush reaction, pharmacology and toxicology

## Abstract

Acetaldehyde is the first active breakdown product (i.e., metabolite) generated during alcohol metabolism. It has toxic properties but also exerts other actions on the body (i.e., has pharmacological properties). Recent studies have shown that the direct administration of acetaldehyde, especially into the brain, induces several effects that mimic those of alcohol. High doses of acetaldehyde induce sedative as well as movement- and memory-impairing effects, whereas lower doses produce behavioral effects (e.g., stimulation and reinforcement) that are characteristic of addictive drugs. When acetaldehyde accumulates outside the brain (i.e., in the periphery), adverse effects predominate and prevent further alcohol drinking. To investigate the role of acetaldehyde in mediating alcohol’s effects, investigators have pharmacologically manipulated alcohol metabolism and the production of acetaldehyde within the body (i.e., endogenous acetaldehyde production). Studies manipulating the activity of the enzyme catalase, which promotes acetaldehyde production in the brain, suggest that acetaldehyde contributes to many behavioral effects of alcohol, especially its stimulant properties. However, it remains controversial whether acetaldehyde concentrations obtained under normal physiological conditions are sufficient to induce significant pharmacological effects. Current evidence suggests that the contribution of acetaldehyde to alcohol’s effects is best explained by a process in which acetaldehyde modulates, rather than mediates, some of alcohol’s effects.

Many chemical compounds, including many medications and drugs, are eliminated from the body through their metabolism, which leads to the production of breakdown products (i.e., metabolites) that are readily excreted. In general, these metabolites are biologically inactive; accordingly, metabolism of the original compound terminates its biological activity. Some metabolites, however, may exert potent effects on the body (i.e., have pharmacological properties) or have toxic properties; these are referred to as active metabolites. Finally, some medications or drugs actually are pharma-cologically inactive compounds; these so-called prodrugs must be converted to biologically active metabolites in order to exert their pharmacological effects.

Acetaldehyde is the first product generated during the metabolism of alcohol (chemically known as ethanol). It is generated primarily in the liver by the enzyme alcohol dehydrogenase (ADH). The acetaldehyde then is converted rapidly to acetate by the enzyme aldehyde dehydrogenase (ALDH). (For more information on the pathways of ethanol metabolism, see the article by Zakhari in this issue.)

Acetaldehyde is an active metabolite that induces a range of toxic, pharmacological, and behavioral effects. However, the role of acetaldehyde in mediating alcohol’s effects, especially its effects on the brain (i.e., its central effects), has been controversial for more than two decades (Deitrich 2004; Quertemont and Tambour 2004). Some investigators argue that acetaldehyde is a key mediator of ethanol’s pharmacological and behavioral effects. According to the most radical version of this theory, ethanol would be a mere prodrug whose effects are fully mediated by its first metabolite, acetaldehyde. It even has been suggested that instead of “alcoholism,” the term “acetaldehydism” would be more appropriate to describe alcohol abuse and addiction (Raskin 1975). Conversely, other scientists deny any significantrole for acetaldehyde in ethanol’s pharmacological effects. These investigators generally contend that following normal alcohol consumption, acetaldehyde concentrations in the blood and brain are far too low to induce any significant pharmacological or behavioral effects (see discussion in Deitrich 2004).

An intermediate, and probably more sustainable, position states that the pharmacological properties of acetaldehyde modulate (rather than mediate) some, but not all, of ethanol’s effects. This modulatory action of acetaldehyde probably greatly depends on specific conditions. For example, acetaldehyde may contribute only to those alcohol effects that occur at high alcohol concentrations, which also result in high acetaldehyde levels. Moreover, the contribution of acetaldehyde to alcohol’s effects likely varies across individuals, in part due to individual differences in alcohol-metabolizing enzymes (Quertemont 2004).

This article provides an overview of acetaldehyde’s pharmacological and behavioral effects in the body and reviews some of the mechanisms that may underlie these effects. It then explores the issue of acetaldehyde concentrations in the brain and periphery before summarizing the results of studies in which ethanol metabolism was manipulated in order to more specifically delineate acetaldehyde’s contribution to ethanol’s effects.

## Acetaldehyde’s Pharmacological and Behavioral Effects

The hypothesis that acetaldehyde mediates or contributes to the effects of ethanol implies that acetaldehyde itself can exert effects similar to those observed after alcohol administration. Therefore, the first step to support such a theory is to demonstrate acetaldehyde’s direct pharmacological and behavioral effects. Because acetaldehyde is highly toxic, however, most studies using direct administration of acetaldehyde have been carried out in laboratory animals, particularly rodents. In humans, most of the knowledge about acetaldehyde’s properties has been gathered indirectly by studying people carrying a deficient variant (i.e., allele) of the gene encoding the ALDH enzyme known as ALDH2*2. This allele results in the production of an inactive ALDH enzyme. If people carrying the deficient ALDH2*2 gene consume alcohol, their bodies cannot metabolize acetaldehyde, which therefore accumulates to high concentrations. Additional information comes from observations of alcoholics who were treated with ALDH inhibitors (e.g., the medication disulfiram) to deter further alcohol consumption but who nevertheless drank alcohol and therefore also accumulated acetaldehyde.

The major problem associated with these observations in humans is the lack of control over acetaldehyde concentrations. Because the bulk of any ingested ethanol is metabolized to acetaldehyde in the liver, genetically or pharmacologically induced deficiencies in ALDH activity lead to high peripheral concentrations of acetaldehyde, allowing no precise determination of the dose-response pattern of acetaldehyde effects. Furthermore, the peripheral effects of these high acetaldehyde levels may mask the compound’s more specific actions in the nervous system (i.e., neuropharmacological properties). Therefore, such studies in humans are not well suited for studying the effects of acetaldehyde in the central nervous system (CNS), particularly the brain.

### Physiological Effects in the Periphery

Acetaldehyde accumulation in the periphery produces a pattern of effects commonly defined by the term “alcohol sensitivity” because these symptoms most often are observed when people with deficient ALDH activity drink alcohol (Eriksson 2001). These typical physiological effects include peripheral widening of the blood vessels (i.e., vasodilation), resulting in increased skin temperature and facial flushing; increased heart and respiration rates; pounding or racing of the heart (i.e., palpitations); lowered blood pressure; narrowing of the airways (i.e., bronchoconstriction); nausea; and headache. The mechanisms by which acetaldehyde induces these symptoms are complex and involve multiple molecular targets, including the following (for a review, see Eriksson 2001):

- Acetaldehyde stimulates the release of signaling molecules called epinephrine and norepinephrine from certain nerve cells (i.e., sympathetic nerve cells) and from a gland located atop the kidneys (i.e., the adrenal gland). These signaling molecules lead to the cardiovascular symptoms of the alcohol sensitivity reaction.
- Acetaldehyde also induces the enhanced release of signaling molecules called histamine and bradykinin, which cause vasodilation and facial flushing.
- Although intermediate acetaldehyde concentrations induce rapid heart beat (i.e., tachycardia) and elevated blood pressure (i.e., hypertension), further increases in acetaldehyde levels lead to abnormally low heart rate and blood pressure, probably because of acetaldehyde’s direct effects on the muscles making up the inner organs (i.e., smooth muscles).

In people with deficient ALDH activity, these peripheral effects together generally lead to an adverse reaction to alcohol and prevent further drinking, thereby reducing these people’s susceptibility to develop alcohol abuse or dependence.

The causal role of acetaldehyde in the alcohol sensitivity reaction has been supported further by studies of people who carry the deficient ALDH2*2 allele or in whom ALDH activity had been pharmacologically inhibited. Investigators treated these people with the compound 4-methylpyrazole—an inhibitor of the ADH enzyme that prevents acetaldehyde production in the periphery. This treatment prevented or reduced the alcohol sensitivity reaction, confirming that acetaldehyde formation is associated with this reaction (Eriksson 2001).

### Behavioral Effects

At the behavioral level, many studies have demonstrated that acetaldehyde is a psychoactive compound whose pattern of effects is similar to that of alcohol (for a review, see Quertemont et al. 2005). At high doses, acetaldehyde induces sedative effects with a loss of consciousness and impaired ability to coordinate movements (i.e., ataxia) with a characteristic straggling gait. It also leads to a significant aversion to any flavor associated with acetaldehyde administration. Moreover, recent studies have indicated that high to intermediate doses of acetaldehyde produce strong memory-impairing (i.e., amnesic) effects in laboratory rodents (Quertemont et al. 2004). The specific effects appear to depend also on the site of administration. Studies in rats found that acetaldehyde stimulates locomotor activity if it is administered directly into the brain (Arizzi-LaFrance et al. 2006) but induces predominantly sedative effects if it is injected in the periphery.

At lower doses, acetaldehyde induces behavioral effects that are characteristic of addictive drugs, such as stimulation and reinforcement. Several studies have focused on the reinforcing properties of acetaldehyde. In humans, there only is anecdotic evidence of these effects. For example, some people who were treated with the ALDH inhibitor disulfiram (which results in acetaldehyde accumulation) reported that they experienced the ethanol–disulfiram interaction and resultant acetaldehyde accumulation as pleasurable (Quertemont 2004). The reinforcing effects of acetaldehyde are better documented in laboratory rats. For example, rats readily self-administer acetaldehyde into the fluid-filled cavities (i.e., ventricles) in the brain, and the voluntary self-administration is much easier to establish for acetaldehyde than for ethanol (Brown et al. 1979). More recently, Rodd-Henricks and colleagues (2002) demonstrated that acetaldehyde is a 1,000-fold more potent reinforcer than ethanol when rats are trained to self-administer these agents into a brain region called the ventral tegmental area, which is strongly involved in ethanol’s reinforcing effects. Finally, Belluzzi and colleagues (2005) found that concurrent administration of acetaldehyde enhanced the acquisition of nicotine self-administration in rats.

Taken together, these findings indicate that with increasing doses, acetaldehyde induces the same biphasic pattern of effects as ethanol on both locomotor activity (stimulation at low doses followed by sedation at high doses) and motivation (reinforcing effects followed by aversion). It also is noteworthy, however, that acetaldehyde does not share all of ethanol’s behavioral properties. For example, in contrast to ethanol, acetaldehyde seems to lack anxiety-reducing (i.e., anxiolytic) properties (Tambour et al. 2005).

### How Does Acetaldehyde Exert Its Behavioral Effects?

The chemical processes in the nervous system (i.e., neurochemical mechanisms) that underlie acetaldehyde’s behavioral effects remain largely unknown. Although several hypotheses have been proposed, to date none of them has been supported by strong experimental evidence. For example, researchers have suggested that acetaldehyde alters various brain signaling mechanisms, including the following (Quertemont et al. 2005):

- Signaling mechanisms involving brain chemicals (i.e., neurotransmitters) known as catecholamines, which include dopamine, epinephrine, and norepinephrine; these neurotransmitters act on peripheral muscles and the heart as well as on the CNS.
- Signaling mechanisms involving brain chemicals called endogenous opioids, which modulate the actions of other neurotransmitters, can induce pain relief and euphoria and contribute to alcohol reinforcement.
- Signaling mechanisms involving the activity of neuronal calcium channels, which are pores in the membrane surrounding nerve cells (i.e., neurons) that can be opened and closed to regulate the levels of calcium ions in the neurons, thereby modifying the excitability of those neurons.

Several studies suggest that acetaldehyde stimulates the activity of a key component of the brain’s reward system, the mesolimbic dopamine system (e.g., Foddai et al. 2004). Most drugs of abuse, including alcohol, stimulate the activity of the mesolimbic dopamine system, and this action is believed to mediate, at least in part, the rewarding effects of these drugs. Therefore, the reinforcing effects of acetaldehyde also may be mediated by activation of this brain system, although further studies are needed to confirm this explanation. Overall, however, evidence supporting any of the mechanisms listed above is rather scarce.

As a highly reactive compound, acetaldehyde can react with many molecules naturally found in the body, including neurotransmitters and proteins (e.g., enzymes), to form new compounds known as adducts that may mediate some of the effects observed after alcohol consumption. Many studies have focused on stable adducts that are formed when acetaldehyde interacts with various proteins. Such acetaldehyde–protein adducts are believed to contribute to the toxic effects associated with chronic alcohol consumption (Freeman et al. 2005). Moreover, certain adducts formed by the reaction of acetaldehyde with catecholamines and other structurally related molecules (e.g., compounds known as indoleamines^1^) have pharmacological effects on the nervous system, including reinforcing properties (see Table). The adducts formed by the interaction of acetaldehyde with catecholamines are called tetrahydroisoquinoline (TIQ) alkaloids, and the adducts formed by the interaction of acetaldehyde with indoleamines are called tetrahydro-β-carboline (THBC) alkaloids.

One of the TIQs that has been extensively studied is salsolinol, which is formed by the reaction of acetaldehyde with dopamine. In addition, acetaldehyde contributes to the accumulation of another TIQ, tetrahydropapaveroline, which is produced by the reaction of dopamine with dopaldehyde, an intermediate in dopamine metabolism. Acetaldehyde inhibits the normal breakdown of dopaldehyde, leading to its accumulation and increased formation of tetrahydropapaveroline. Both salsolinol and tetrahydropapaveroline exhibit reinforcing properties and induce a long-lasting increase in voluntary alcohol consumption in rodents and monkeys (Quertemont et al. 2004). Both compounds therefore are believed to contribute to the development of alcoholism; however, their neurochemical mechanisms of action remain unknown. Similarly, it is unclear whether the concentrations of these TIQ and THBC alkaloids that are achieved in the brain after alcohol consumption are pharmacologically relevant. The answers to these questions are critical for determining whether these acetaldehyde adducts do indeed play a role in the neuropharmacological effects of alcohol.

In summary, acetaldehyde is a pharmacologically active compound that acts either directly or through the formation of adducts to induce effects in both the periphery and the brain. Of particular interest, some of the behavioral effects of acetaldehyde are similar to those of ethanol, leading to the suggestion that acetaldehyde may be involved in mediating these effects of alcohol. For example, a growing body of evidence indicates that acetaldehyde shows reinforcing properties. Therefore, it has been speculated that acetaldehyde contributes to the motivation to drink alcohol and, consequently, to the development of alcoholism (Brown et al. 1979; Rodd-Henricks et al. 2002). However, it is far too early to claim that acetaldehyde shares all of ethanol’s properties, and further studies are required to better characterize the effects of direct acetaldehyde administration and to identify the molecular targets that mediate these effects.

## Physiological Acetaldehyde Concentrations—A Controversial Issue

Although acetaldehyde unquestionably is an active compound with both pharmacological and toxic properties, researchers have not been able to conclusively establish that acetaldehyde can induce these effects in the living organism (i.e., in vivo) at the physiological concentrations obtained after alcohol consumption. Although this question has long been debated, controversy still exists. After alcohol ingestion, acetaldehyde mainly is produced during ethanol breakdown in the liver, which primarily involves the enzyme ADH but also cytochrome P4502E1 and the enzyme catalase. Because of the high efficiency of the liver ALDH, however, acetaldehyde is rapidly converted to acetate and little acetaldehyde reaches the blood circulation. Therefore, under normal physiological conditions, acetaldehyde concentrations in the blood following alcohol administration usually are very low or even undetectable. Higher concentrations of circulating acetaldehyde have been reported only in chronic alcohol consumers and in people carrying the deficient ALDH2*2 allele.

Because acetaldehyde must act on the brain to induce behavioral effects, the physiological acetaldehyde concentrations in the brain and other organs also have been investigated. ADH, the main ethanol-metabolizing enzyme, is not physiologically active in the brain, and researchers long have assumed that the CNS cannot metabolize alcohol and produce acetaldehyde. However, recent findings suggest that the brain can produce acetaldehyde from local ethanol metabolism involving mainly catalase and cytochrome P4502E1 (Zimatkin et al. 2006). Moreover, studies conducted with cultured cells (i.e., in vitro studies) have indicated that pharmacologically significant acetaldehyde concentrations should be obtainable in the brain following ethanol administration (Zimatkin and Deitrich 1997). To date, however, it has not been unambiguously shown that brain acetaldehyde concentrations are high enough in vivo to contribute significantly to ethanol’s effects on the brain. This failure also may be due to the fact that acetaldehyde concentrations after ethanol administration differ among brain regions because the acetaldehyde-producing enzymes are not evenly distributed across various brain cells (Zimatkin and Lindros 1996). It is therefore possible that past attempts to measure brain acetaldehyde concentrations underestimated its potential neurochemical actions. Moreover, it is possible that although low acetaldehyde concentrations themselves have no measurable effects, they may suffice to synergistically enhance the effects of ethanol.

In summary, it remains unclear whether the acetaldehyde concentrations achieved in different organs, especially in the brain, after alcohol consumption under normal physiological conditions are biologically relevant. Finding the answer to this question will be critical for definitively determining whether acetaldehyde contributes to the effects of ethanol in vivo. Another strategy to determining whether acetaldehyde mediates or modulates the effects of ethanol is to modify physiological acetaldehyde concentrations by interfering with normal ethanol metabolism. This approach is described in the next section.

## Effects of Altering Ethanol Metabolism

Although direct administration of acetaldehyde to an organism can show researchers what effects acetaldehyde can have at the sometimes very high concentrations achieved, such experiments do not reflect acetaldehyde’s actual effects during alcohol intoxication (Deitrich 2004). To test the hypothesis that acetaldehyde mediates or modulates ethanol’s effects, researchers instead have sought to modify the acetaldehyde concentrations that result from endogenous ethanol metabolism after alcohol administration and then to assess the consequences of this manipulation on ethanol’s effects. To this end, several animal studies have used pharmacological agents that alter normal ethanol metabolism.

As mentioned earlier, the bulk of any ingested ethanol is metabolized to acetaldehyde by liver ADH; nevertheless, manipulation of ADH activity usually is not a useful experimental strategy for studying the role of acetaldehyde in alcohol’s effects for several reasons:

- In the CNS, ADH is not physiologically active and brain acetaldehyde concentrations therefore do not depend on ADH activity.
- In the periphery, the high efficiency of liver ALDH prevents acetaldehyde produced in the liver from escaping into the blood circulation; as a result, changes in ADH activity do not significantly alter blood acetaldehyde concentrations if ALDH is not inhibited at the same time.

**Figure 1:**
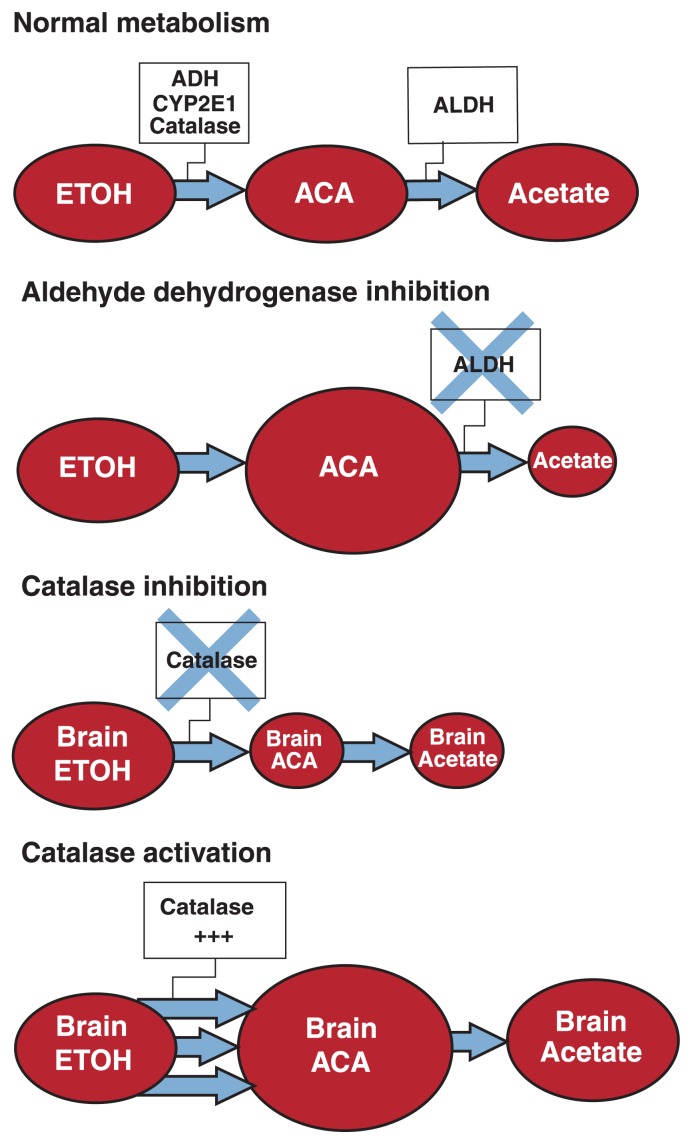
Schematic representation of the metabolism of ethanol (ETOH) and the effects of aldehyde dehydrogenase (ALDH) inhibitors and catalase modulators. Under normal physiological conditions, ethanol is metabolized to acetaldehyde (ACA) through several enzymatic pathways involving alcohol dehydrogenase (ADH), cytochrome P4502E1 (CYP2E1), or catalase. When ALDH is pharmacologically inhibited, acetaldehyde accumulates to high concentrations both in the brain and in the periphery. Catalase metabolizes about 60 percent of ethanol in the brain. Therefore, inhibition of catalase is believed to reduce brain acetaldehyde levels, whereas enhancement of catalase activity is believed to increase brain acetaldehyde levels.

To circumvent these problems, changes in peripheral or CNS acetaldehyde concentrations are typically achieved by modulating the activity of ALDH and of the acetaldehyde-producing enzyme catalase (see Figure 1). Several ALDH inhibitors have been used to cause massive acetaldehyde accumulation after alcohol consumption, most commonly disulfiram and cyanamide. Because catalase is believed to account for most of the acetaldehyde production in the brain, various modulators of its activity have been tested to specifically assess the contribution of acetaldehyde to ethanol’s effects on the brain.

### Effects of ALDH Inhibition

Ethanol administration to animals or humans following treatment with ALDH inhibitors leads to the typical alcohol sensitivity reaction, which then deters further alcohol consumption. Accordingly, most animal studies using ALDH inhibitors have focused on measuring subsequent alcohol consumption in order to establish a model for predicting the efficacy of ALDH inhibitors as alcohol-deterrent medications in alcoholism treatment. These studies generally concluded that ALDH inhibition and acetaldehyde accumulation strongly reduce voluntary alcohol consumption and potentiate the aversion for moderate to high ethanol doses (Quertemont et al. 2005).

Another approach to interfering with ALDH activity was used by Isse and colleagues (2005), who generated mice that no longer produced active ALDH2 (i.e., ALDH2 knockout mice) because the function of the gene that controls ALDH2 production was altered in these animals. As a result of the manipulation, these mice lack active ALDH in the liver and therefore eliminate acetaldehyde at a very low rate. Like humans carrying the deficient ALDH2*2 allele, these mice showed higher blood acetaldehyde concentrations after alcohol administration. They also displayed the typical symptoms of the alcohol sensitivity reaction, such as redness of the skin (i.e., the typical flushing reaction found in humans). Additionally, the ALDH2 knockout mice avoided voluntary alcohol consumption, confirming that high blood acetaldehyde levels induce adverse effects that prevent alcohol consumption. Together with the studies using ALDH inhibitors, these findings suggest that acetaldehyde may contribute to the aversive effects of high ethanol doses.

An important disadvantage of these studies, however, is the lack of control over acetaldehyde concentrations. Indeed, ethanol administration to animals or humans pretreated with ALDH inhibitors leads to peripheral acetaldehyde concentrations that are substantially higher than the normal range of physiological concentrations. This limitation makes interpretations in terms of acetaldehyde contribution to the effects of ethanol difficult.

### Effects of Manipulation of Catalase Activity

A second strategy that has been widely used to unravel the contribution of acetaldehyde to the central effects of ethanol is based on pharmacological manipulations of catalase activity. Catalase plays an important role in acetaldehyde production in the brain, and manipulations of catalase levels were shown to alter acetaldehyde concentrations when brain tissue studied in vitro was treated with ethanol (Smith et al. 1997). Similarly, catalase inhibition is expected to decrease brain acetaldehyde concentrations, and catalase activation is expected to increase brain acetaldehyde levels after ethanol administration in vivo. However, catalase only marginally contributes to ethanol metabolism in the liver, and experimental manipulation of catalase activity therefore should have no significant effects on peripheral acetaldehyde levels.

Several studies conducted in mice have investigated the role that acetaldehyde and its production by catalase play in ethanol’s locomotor stimulant effects. The results of these studies generally are consistent with the idea that acetaldehyde contributes to the stimulant effects of ethanol (Quertemont et al. 2005). For example, various treatments resulting in inhibition of catalase activity reduced the locomotor stimulant effects of ethanol (e.g., Escarabajal et al. 2000). Conversely, the potentiation of catalase activity enhanced ethanol-induced locomotion (e.g., Correa et al. 2005). Consistent with these findings, researchers observed that mice which exhibit a 60percent reduction in brain catalase activity compared with normal mice showed a reduced sensitivity to the locomotor stimulant effects of ethanol (Aragon et al. 1992).

Pharmacological inhibition of catalase activity also led to a reduction in a range of behavioral effects of ethanol (e.g., ethanol-induced sedation, aversion, and memory impairment) and increased the dose at which ethanol was lethal to the animals (Smith et al. 1997). Finally, several studies have investigated the role of acetaldehyde in the motivational and reinforcing effects of ethanol by evaluating the effects of catalase inhibitors and activators on various indicators of voluntary alcohol consumption in rodents (Aragon and Amit 1992; He et al. 1997). However, these studies have yielded conflicting results that are difficult to reconcile, as have studies investigating the relationship between brain catalase activity and the natural propensity to drink alcohol in rodents. Therefore, it is difficult to conclude from the catalase studies conducted to date if and how brain acetaldehyde levels impact ethanol’s motivational and reinforcing effects.

Thus, although studies on brain catalase activity suggest that acetaldehyde might be involved in or even mediate some of ethanol’s behavioral effects, particularly its stimulant effects, the role of acetaldehyde in the motivational and reinforcing properties of alcohol remains inconclusive. Furthermore, all of the catalase-modulating studies suffer from several important weaknesses. First, because brain acetaldehyde levels are difficult to measure in vivo, these studies did not attempt to measure the effects of their experimental treatments on acetaldehyde concentrations but instead only resorted to making assumptions on the effectiveness of their manipulations (Deitrich 2004). In particular, the effects of catalase activation on brain acetaldehyde concenrations as depicted in Figure 1 remain speculative and have not been experimentally demonstrated. Second, most of the pharmacological agents that are commonly used to alter catalase activity have a poor specificity—that is, they also interfere with other physiological reactions (Quertemont et al. 2005). As a result, alternative explanations for the observed effects that do not involve acetaldehyde often are possible. Therefore, the role of acetaldehyde in the observed effects remains hypothetical, and caution should be used when interpreting the results of catalase studies as evidence of the contribution of acetaldehyde to ethanol’s effects.

## Conclusions

Acetaldehyde is an active metabolite with a range of toxic and pharmacological effects, and many of the effects induced by direct acetaldehyde application mimic those of ethanol. In particular, administration of low doses of acetaldehyde to the brain produces behavioral effects that are typical of addictive drugs, such as psychostimulation and reinforcement. In contrast, accumulation of high acetaldehyde levels in the periphery leads to a strong alcohol aversion and prevents further alcohol drinking.

The contribution of such acetaldehyde-induced effects to the overall effects of alcohol consumption under normal physiological conditions still is controversial. The main issue in these discussions is the acetaldehyde concentration that typically is achieved after alcohol consumption in vivo, under normal physiological conditions. Nevertheless, studies involving alteration of catalase activity provide, despite their obvious weaknesses, converging evidence that acetaldehyde contributes to various behavioral effects of ethanol, especially its stimulant properties.

**Figure 2:**
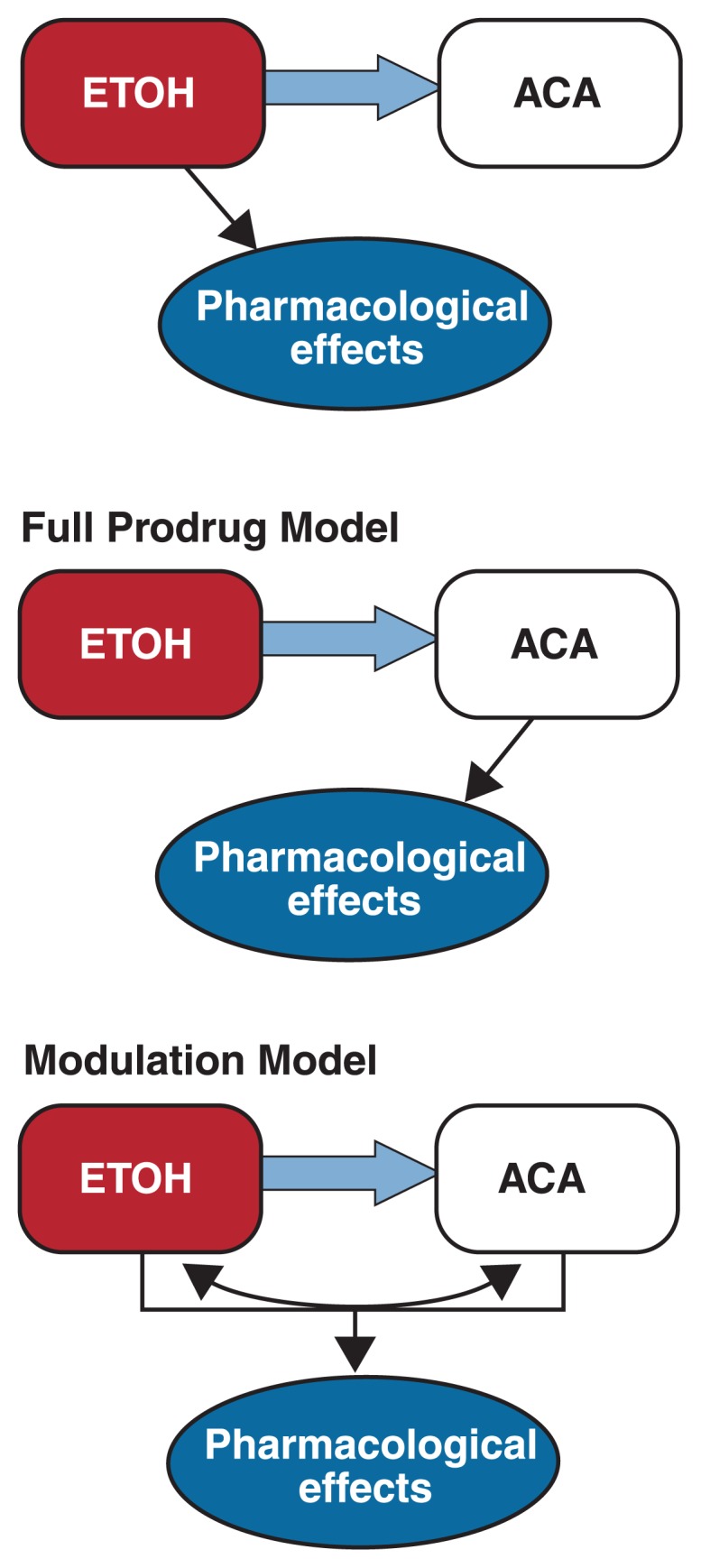
Schematic representation of three alternative models that account for the role of acetaldehyde in ethanol’s (ETOH’s) effects. According to the ethanol model, acetaldehyde (ACA) does not contribute at all to ethanol’s overall pharmacological effects, and all effects are mediated directly by the molecular action of ethanol. The full prodrug model states that all pharmacological effects of ethanol are mediated by acetaldehyde. According to this model, ethanol would be a mere prodrug without pharmacological effect of its own. Finally, the intermediate modulation model stipulates that acetaldehyde synergistically interacts with ethanol to modulate ethanol’s pharmacological effects.

Three alternative models regarding the contribution of acetaldehyde to ethanol’s effects have been put forward (see Figure 2):

- The ethanol model posits that acetaldehyde does not contribute at all to the pharmacological effects of ethanol. This model is mainly based on the contention that the in vivo concentrations of acetaldehyde in target organs are insufficient to induce significant pharmacological actions.
- The full prodrug model contends that acetaldehyde mediates all of the pharmacological effects of ethanol.
- The modulation model states that the pharmacological actions of acetaldehyde modulate some of ethanol’s effects.

Whereas the full prodrug model seems to be least likely, it currently is difficult to decide between the other two models. The modulation model appears to best account for the results of the studies using acetaldehyde administration or modulation of catalase activity. However, it is possible and even likely that the three models coexist for different effects of ethanol. For example, whereas acetaldehyde might not be involved at all in ethanol’s anxiolytic effects (Tambour et al. 2005), it could mediate ethanol’s stimulant properties. Further studies, especially in vivo assessments of acetaldehyde concentrations, clearly are needed to clarify the role of acetaldehyde in the effects of alcohol consumption. Only when the actual acetaldehyde concentrations found in vivo in various organs following alcohol consumption are known can reliable conclusions on the involvement of acetaldehyde in ethanol’s effects be drawn.

## Figures and Tables

**Table:** Chemical Structures of the Main Condensation Products of Acetaldehyde With Endogenous Biogenic Amines

Biogenic Amines Condensing With Acetaldehyde	Name of Condensation Product	Chemical Structure
Dopamine	6,7-dihydroxy-1-methyl-1,2,3,4-tetrahydroisoquinoline (Salsolinol)	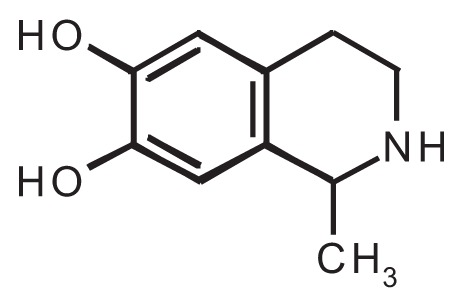
Serotonin	6-hydroxy-1-methyl-1,2,3,4-tetrahydro-β-carboline (6-OH-MTBC)	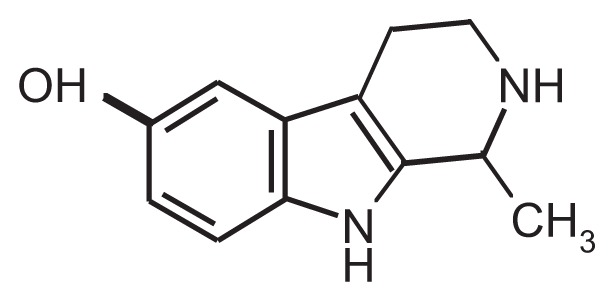
Tryptamine	1-methyl-1,2,3,4-tetrahydro-β-carboline (MTBC)	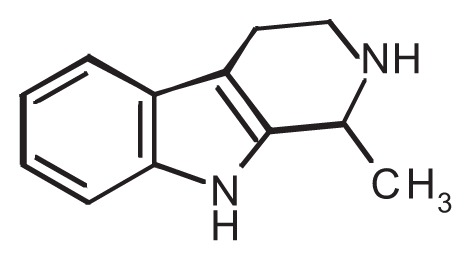
Tryptophan	3-carboxy-1-methyl-1,2,3,4-tetrahydro-β-carboline (3-MTBC)	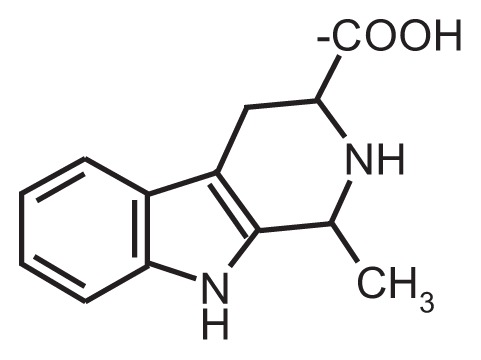

## Footnotes

1 Both catecholamines and indoleamines belong to the group of biogenic amines, which are organic compounds formed during biochemical processes in plants and animals that carry a nitrogen atom as a central molecule.
